# Low frequency of *AIP* mutations in patients with young-onset sporadic pituitary macroadenomas

**DOI:** 10.1007/s40618-023-02083-7

**Published:** 2023-05-07

**Authors:** L. M. Gaspar, C. I. Gonçalves, C. Saraiva, L. Cortez, C. Amaral, E. Nobre, M. C. Lemos

**Affiliations:** 1 CICS-UBI, Health Sciences Research Centre, University of Beira Interior, 6200-506 Covilhã, Portugal; 2 Serviço de Endocrinologia, Hospital de Egas Moniz, Centro Hospitalar Lisboa Ocidental, Lisbon, Portugal; 3 Serviço de Endocrinologia, Hospital de Curry Cabral, Centro Hospitalar Universitário Lisboa Central, Lisbon, Portugal; 4 Serviço de Endocrinologia, Hospital de Santo António, Centro Hospitalar Universitário do Porto, Porto, Portugal; 5 Serviço de Endocrinologia, Diabetes e Metabolismo, Hospital de Santa Maria, Centro Hospitalar Universitário Lisboa Norte, Lisbon, Portugal

**Keywords:** Pituitary adenoma, AIP, Aryl hydrocarbon receptor interacting protein, Mutation

## Abstract

**Purpose:**

Mutations in the aryl hydrocarbon receptor interacting protein (*AIP*) gene cause familial isolated pituitary adenomas (FIPA). *AIP* mutations have also been found in patients with apparently sporadic pituitary adenomas, particularly in young patients with large adenomas. The aim of this study was to determine the frequency of *AIP* germline mutations in patients with young-onset sporadic pituitary macroadenomas.

**Methods:**

The *AIP* gene was sequenced in 218 Portuguese patients with sporadic pituitary macroadenomas diagnosed before the age of 40 years.

**Results:**

Heterozygous rare sequence variants in *AIP* were identified in 18 (8.3%) patients. However, only four (1.8%) patients had pathogenic or likely pathogenic variants. These consisted of two already known mutations (p.Arg81* and p.Leu115Trpfs*41) and two novel mutations (p.Glu246*, p.Ser53Thrfs*36). All four patients had GH-secreting adenomas diagnosed between the ages of 14 and 25 years. The frequency of *AIP* pathogenic or likely pathogenic variants in patients under the age of 30 and 18 years was 3.4% and 5.0%, respectively.

**Conclusion:**

The frequency of *AIP* mutations in this cohort was lower than in other studies. Previous reports may have overestimated the contribution of *AIP* mutations due to the inclusion of genetic variants of uncertain significance. The identification of novel *AIP* mutations expands the known spectrum of genetic causes of pituitary adenomas and may help understand the role of *AIP* mutations in the molecular mechanisms underlying pituitary tumorigenesis.

## Introduction

The vast majority of pituitary adenomas occur in a sporadic context and are likely to occur due to acquired somatic and epigenetic mutations [1, 2]. More rarely, pituitary adenomas occur as part of syndromic diseases (e.g. multiple endocrine neoplasia) or as Familial Isolated Pituitary Adenomas (FIPA), due to inherited germline mutations [3].

Germline mutations in the Aryl Hydrocarbon Receptor-Interacting Protein (*AIP*) gene have been identified in cases of FIPA [4]. The *AIP* gene comprises six exons and encodes a 330-amino acid co-chaperone protein, which has a role in the metabolic clearance of dioxin and other toxic carcinogenic agents and in the regulation of the cyclic adenosine monophosphate (cAMP) molecular pathway [5, 6]. The expression of *AIP* in pituitary cells reduces cell proliferation rate, suggesting that *AIP* may act as a tumour suppressor gene [7]. Heterozygous inactivating mutations in *AIP* have been reported along the entire coding region of the gene [8]. Tumours that arise in individuals with *AIP* mutations usually present at a younger age, have a larger size, show increased invasiveness, and are often resistant to standard treatments [1, 9]. Prospective studies have shown that the identification of patients with *AIP* mutations may result in improved clinical outcomes [10].

Due to incomplete penetrance, patients with *AIP* mutations may not have a recognized family history of pituitary adenomas and may be classified as sporadic cases. Studies of patients with apparently sporadic pituitary adenomas have demonstrated that a variable proportion of these are due to germline *AIP* mutations. The prevalence of *AIP* mutations has been reported to be around 3.6% in unselected patients with sporadic pituitary adenomas [11]. However, the prevalence of *AIP* mutations increases to 7.2% in patients diagnosed under the age of 40 years [11], to 11.7% in patients with macroadenomas diagnosed under the age of 30 years [12], and to over 20% in paediatric patients [11, 12]. These findings have led to recommendations for *AIP* mutation testing in patients with pituitary macroadenomas diagnosed under the age of 30 [12] or 40 years [11].

The aim of this study was to investigate the prevalence of *AIP* mutations in a large Portuguese cohort of young-onset sporadic pituitary macroadenomas.

## Materials and methods

### Subjects

This multicentre study involved 218 Portuguese patients recruited consecutively at the main endocrinology outpatient clinics in Portugal, from 2018 to 2022. Selection criteria were patients with sporadic macroadenomas (tumour greater diameter ≥ 1 cm) diagnosed under the age of 40 years. Patients with a family history of pituitary adenomas (i.e. an affected first or second degree family member) or with evidence of a syndromic form of pituitary adenomas were excluded. Mean age (± standard deviation) at diagnosis was 28.9 ± 7.5 years, 118 patients were under 30 years at diagnosis, and 20 patients were under 18 years at diagnosis. Gender distribution was 113 (52%) females and 105 (48%) males. The type of adenoma was based on histological examination, or in the case of prolactinomas, by clinical, hormonal and radiological examination. Eighty (36.7%) patients had prolactinomas, 61 (28.0%) had somatotropinomas, 34 (15.6%) had non-functioning pituitary adenomas; 14 (6.4%) had corticotropinomas, 15 (6.9%) had mixed-secreting pituitary adenomas; seven (3.2%) had gonadotropinomas, one had a thyrotropinoma, and six (2.8%) had adenomas with undetermined histology. Written informed consent was obtained from all subjects and the study was approved by the Ethics Committee of the Faculty of Health Sciences, University of Beira Interior (Ref. CE-UBI-Pj-2018-027).

### Genetic analysis

Genomic deoxyribonucleic acid (DNA) from each patient was extracted from peripheral blood leukocytes using previously described methods [13]. Patients were screened for mutations in *AIP* by Polymerase Chain Reaction (PCR) amplification of the six coding exons and exon–intron boundaries and through bi-directional sequencing using a semi-automated DNA sequencer (STAB VIDA, Caparica, Portugal; and ABI 3730XL, Applied Biosystems; Thermo Fisher Scientific, Waltham, MA, USA). Primer sequences were previously described by Cazabat et al. [11]. Genomic sequence variants were selected according to the following cumulative criteria: (1) Located in the *AIP* exons and up to 10 nucleotides from the splice site regions; and (2) Absent or rare (population frequency < 1%) in the Genome Aggregation Database (gnomAD) [14]. Selected variants were then classified by the American College of Medical Genetics and Genomics (ACMG) criteria [15], using the VarSome search engine [16]. Variants classified as pathogenic or likely pathogenic were considered to be causative mutations. The heterozygous frameshift mutations were confirmed by cloning of the PCR product using a CloneJET PCR Cloning Kit (ThermoScientific, Thermo Fisher Scientific, Waltham, MA, USA) followed by DNA sequencing of each allele. Sequence variant nomenclature followed standard guidelines [17] and was based on the cDNA reference sequence for the *AIP* gene (GenBank accession NM_003977.4). The frequency of *AIP* mutations was determined in patients diagnosed under the ages of 40, 30 and 18 years, respectively.

## Results

### Genetic findings

**Fig. 1:**
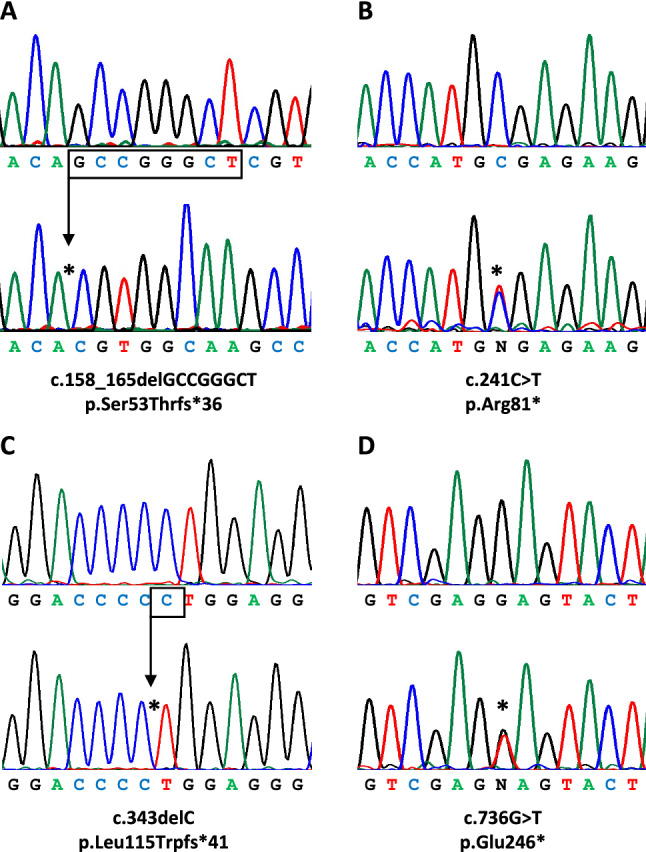
*AIP* mutations identified in patients. For each mutation (**A**–**D**), the DNA sequences of a normal individual (above) and the patient (below) are shown. The positions of the mutations are indicated by asterisks. All mutations were heterozygous. For clarity, the deletion mutations (**A**, **C**) are shown only on the cloned sequences of the mutated alleles

Sequence analysis of the entire coding region of *AIP*, including the exon–intron boundary regions, revealed that 18 (8.3%) patients had 12 different heterozygous rare sequence variants. However, eight of these variants [c.47G > A (p.Arg16His), c.132C > T (p.Asp44Asp), c.753G > A (p.Leu251Leu), c.891C > A (p.Ala297Ala), c.896C > T (p.Ala299Val), c.*14C > A, c.*60G > C and c.*64G > C] were classified as benign according to ACMG criteria (Table 1). The remaining four variants [c.158_165delGCCGGGCT (p.Ser53Thrfs*36), c.241C > T (p.Arg81*), c.343delC (p.Leu115Trpfs*41), and c.736G > T (p.Glu246*)] were classified as pathogenic or likely pathogenic (Table 1, Fig. 1). Thus, 1.8% (4/218) of the patients with sporadic pituitary macroadenomas were considered to have causative mutations in *AIP*. All four patients with *AIP* mutations had been diagnosed before the age of 30 years. In patients diagnosed under the age of 30, the prevalence of *AIP* mutations in this group was 3.4% (4/118). In the paediatric age group (< 18 years-old), the prevalence of *AIP* mutations was 5.0% (1/20).

**Table 1:** Clinical and genetic characteristics of patients with *AIP* rare sequence variants

Patient number	Sex	Age at diagnosis (yr)	Type of adenoma	Size of adenoma (mm)	Variant (nucleotide, protein level) (a)	GnomAD allele frequency	ACMG classification (criteria) (b)	Previously reported
*Pathogenic and likely pathogenic variants*								
1	F	20	GH-secreting	20	c.158_165delGCCGGGCT, p.Ser53Thrfs*36	0	LP (PVS1, PM2)	No
2	M	22	GH-secreting	26	c.241C>T, p.Arg81*	0	P (PVS1, PP5, PM2)	Yes [7]
3	M	14	GH/PRL-secreting	14	c.343delC, p.Leu115Trpfs*41	0	LP (PVS1, PM2)	Yes [28]
4	F	25	GH-secreting	28	c.736G>T, p.Glu246*	0	LP (PVS1, PM2)	No
*Benign variants*								
5	M	18	PRL-secreting	>10	c.47G>A, p.Arg16His	0.0020820	B (BS1, BS2, BP1, BP4, BP6)	Yes [35]
6	F	20	GH-secreting	34	c.132C>T, p.Asp44Asp	0.0079840	B (BA1, BP4, BP6, BP7)	Yes [36]
7	M	33	GH/PRL/FSH/LH-secreting	>10	c.132C>T, p.Asp44Asp	0.0079840	B (BA1, BP4, BP6, BP7)	Yes [36]
8	F	37	PRL-secreting	20	c.132C>T, p.Asp44Asp	0.0079840	B (BA1, BP4, BP6, BP7)	Yes [36]
9	F	40	PRL-secreting	31	c.132C>T, p.Asp44Asp	0.0079840	B (BA1, BP4, BP6, BP7)	Yes [36]
10	F	40	GH-secreting	27	c.753G>A, p.Leu251Leu	0.0001757	B (BS1, BS2, BP4, BP7)	Yes [37]
					c.*14C>A	0.0004049	B (BS1, BS2, BP4)	
11	F	28	ACTH-secreting	11	c.891C>A, p.Ala297Ala	0.0018130	B (BS1, BS2, BP4, BP6, BP7)	Yes [18, 38]
					c.*64G>A	0.0054470	B (BS1, BS2, BP4, BP6)	
12	F	17	PRL-secreting	>10	c.896C>T, p.Ala299Val	0.0005441	B (BS1, BS2, BP1, BP4)	Yes [39]
13	F	26	PRL-secreting	12	c.896C>T, p.Ala299Val	0.0005441	B (BS1, BS2, BP1, BP4)	Yes [39]
14	F	30	PRL-secreting	15	c.896C>T, p.Ala299Val	0.0005441	B (BS1, BS2, BP1, BP4)	Yes [39]
15	F	36	ACTH-secreting	14	c.896C>T, p.Ala299Val	0.0005441	B (BS1, BS2, BP1, BP4)	Yes [39]
16	M	37	GH-secreting	>10	c.896C>T, p.Ala299Val	0.0005441	B (BS1, BS2, BP1, BP4)	Yes [39]
17	M	28	PRL-secreting	>40	c.*60G>C	0.0078700	B (BS1, BS2, BP4, BP6)	Yes [40]
18	M	29	PRL-secreting	26	c.*60G>C	0.0078700	B (BS1, BS2, BP4, BP6)	Yes [40]

F, female; M, male; yr, years; ACTH, adrenocorticotropic hormone; FSH, follicle stimulating hormone; GH, growth hormone; LH, luteinizing hormone; PRL, prolactin; mm, millimeters; GnomAD, Genome Aggregation Database. (a) All variants were heterozygous and nucleotide numbering was based on coding reference sequence NM_003977.4. (b) American College of Medical Genetics and Genomics (ACMG) classification of variants (P, pathogenic; LP, likely pathogenic; B, benign) was based on the evidence for pathogenicity [very strong (PVS1), moderate (PM1–6), or supporting (PP1–5)] or benign impact [stand-alone (BA), strong (BS1-4), or supporting (BP1-7)]. ACMG classifications were based on the VarSome search engine (https://varsome.com/), accessed on 27 December 2022

### Clinical characteristics of patients with *AIP* mutations

#### Patient 1

A 20-years-old woman presented with secondary amenorrhea. The amenorrhea had been attributed to anorexia nervosa diagnosed at the age of 15, but did not reverse with the recovery of body weight. She also complained of frontal headaches, excessive sweating and coarse facial features. She denied any visual defects. Her height and weight was 167 cm and 59 kg, respectively. Blood tests revealed basal growth hormone (GH) 14 ng/mL (normal range (NR) < 8), insulin-like growth factor 1 (IGF1) 1100 ng/mL (NR 102–231), follicle-stimulating hormone (FSH) 5.4 mUI/mL (NR 1.2–9), luteinizing hormone (LH) 2.1 mUI/mL (NR 1.1–11.6), estradiol < 5 pg/mL, prolactin 45 ng/mL (NR 4.7–23.0), adrenocorticotropic hormone (ACTH) 30 pg/mL (NR 7.2–63.3), cortisol 15.1 μg/dL (NR 6.2–18.0), thyroid-stimulating hormone (TSH) 1.6 mUI/mL (NR 0.3–4.2) and free thyroxine (FT4) 1.1 ng/dL (NR 0.85–1.7). An oral glucose tolerance test (OGTT) demonstrated failure of GH suppression, with a GH nadir of 1.4 ng/mL. A magnetic resonance imaging (MRI) showed a 20 mm pituitary tumour. A diagnosis of acromegaly was made and the patient underwent transsphenoidal surgery with excision of the pituitary macroadenoma. The histopathology showed a pituitary adenoma with a predominance of acidophilic cells. Post-surgical testing revealed normalization of GH levels, but persistence of the hypogonadotropic hypogonadism. At the age of 29, she underwent fertility treatment and had a twin pregnancy. At the last follow-up, at the age of 37, blood tests revealed basal GH 0.42 ng/mL (NR < 8), IGF1 102 ng/mL (NR 102–231), FSH 1.4 mUI/mL (NR 1.2–9), LH 0.16 mUI/mL (NR 1.1–11.6), estradiol < 5 pg/mL, prolactin 14.8 ng/mL (NR 4.7–23.0), ACTH 22.2 pg/mL (NR 7.2–63.3), cortisol 16.1 μg/dL (NR 6.2–18.0), TSH 0.95 mUI/mL (NR 0.3–4.2) and FT4 1.2 ng/dL (NR 0.85–1.7). There was no known family history of pituitary adenomas. Genetic analysis revealed an *AIP* heterozygous mutation (c.158_165delGCCGGGCT, p.Ser53Thrfs*36) in the patient. No other family members were available for genetic screening.

#### Patient 2

A 23-year-old man presented to the emergency department with a history of frequent and severe headaches, without visual defects. An MRI showed a 20 × 18 mm pituitary tumour with suprasellar extension contacting the optic chiasm and evidence of bleeding into the tumour. Acral growth and prognathism were noted. His height and weight were 189 cm and 97 kg, respectively. Serum IGF1 level was 704 ng/mL (NR 115–340), basal GH level was 6.40 ng/mL and prolactin was 17.40 ng/mL (NR 4.04–15.20), whereas FT4, TSH, cortisol and electrolyte levels were within the normal range. An OGTT demonstrated failure of GH suppression with a GH nadir of 5.82 ng/mL. A diagnosis of acromegaly was made and he was proposed for transsphenoidal surgery. In the meantime, he presented to the emergency department with refractory headaches, visual deficit and ptosis. MRI showed a 24 × 22 × 26 mm tumor with suprasellar and cavernous extension and acute bleeding in the tumour. The patient underwent transsphenoidal surgery and histopathology showed a pituitary adenoma with frequent immunoreactive cells for GH and rarer for prolactin and with areas of recent and old intratumoral haemorrhage, suggestive of pituitary apoplexy. Post-surgical testing revealed cortisol and thyroxine deficiencies that were corrected with hydrocortisone 15 mg/day and levothyroxine 50 ug/day. At the last follow-up, 8 months after surgery, prolactin and testosterone levels were normal, IGF1 level was 127 ng/mL (NR 115–340) and the nadir postglucose GH level was 0.1 ng/mL. The patient began reducing the dosages of the replacement therapy. There was no known family history of pituitary adenomas. Genetic analysis revealed an *AIP* heterozygous mutation (c.241C > T, p.Arg81*) in the patient and in his asymptomatic father, who was referred for clinical, biochemical and MRI assessment.

#### Patient 3

A 13-year-old boy presented with tall stature. Since the age of 8 years, he demonstrated increased height velocity and started to cross height percentiles and remained above the 97th percentile. The patient also complained of frontal headaches for 2 years. He denied any visual defects. His height and weight was 180.8 cm (+ 3.3 SDS) and 57 kg, respectively. He displayed coarse facial features with frontal bossing and mild prognathism, large hands and feet. His pubertal status was compatible with Tanner stage 5 (testicular volume 20 mL). Past medical history was unremarkable. The mid-parental height was 178.5 cm. Baseline pituitary investigation showed increased levels of IGF1 (587.5 ng/mL, NR 74–450), random GH 11.3 ng/mL (NR 0.12–8.1), hyperprolactinaemia with PRL 35.1 ng/mL (NR 4.4–19), central hypothyroidism with TSH 2.3 µUI/mL (NR 0.51–4.30) and FT4 6.96 pmol/L (NR 12.6–21), morning cortisol of 10.3 µg/dL (NR 6.2–12.5), total testosterone 238 ng/dL (NR 105–545), FSH 3.49 U/L (NR 1.5–8.6), LH 1.64 U/L (NR 1.7–8.6). An OGTT demonstrated failure of GH suppression, with a GH nadir of 8.1 ng/mL. MRI of the sellar region showed a pituitary tumour, measuring 13 × 12 × 14 mm, without signs of cavernous sinus invasion, suprasellar extension or optic chiasm compression. Visual field tests were normal. Bone age was coincident with his chronological age. A diagnosis of pituitary gigantism due to a GH-secreting pituitary macroadenoma and associated central hypothyroidism was made. Treatment with levothyroxine was initiated and the patient underwent transsphenoidal surgery with excision of the pituitary macroadenoma, without complications. Histopathology revealed a mammosomatotroph adenoma with a Ki-67 index of < 1%. Three months after surgery, he presented normalisation of IGF1 levels, GH nadir during OGTT was < 1 ng/mL and no residual tumour was found on the MRI. Recovery of central hypothyroidism was also observed after surgery, and the patient is currently euthyroid without levothyroxine supplementation. He remains in remission 7 years after surgery with serum IGF1 of 308 µg/L (NR 93–449), nadir GH level < 1 ng/mL during OGTT, and a height of 189.5 cm. There was no known family history of pituitary adenomas. Genetic analysis revealed an *AIP* heterozygous mutation (c.343delC, p.Leu115Trpfs*41) in the patient. No other family members were available for genetic screening.

#### Patient 4

A 25-year-old woman presented with acromegalic facial features, acral growth and secondary amenorrhea. Her height and weight were 159 cm and 61 kg, respectively. Blood tests revealed basal GH 29.8 ng/mL (NR 0.06–5.0), IGF1 895 ng/mL (NR 117–329), FSH 3.2 mUI/mL (NR 1.2–9), LH 1.69 mUI/mL (NR 1.1–11.6), estradiol 15 pg/mL (NR 15–160), prolactin 42 ng/mL (NR 3.46–19.4), ACTH 30.5 pg/mL (NR 5–46), cortisol 9.8 μg/dL (NR 5–25), TSH 1.95 mUI/mL (NR 0.4–4.0) and FT4 0.8 ng/dL (NR 0.8–1.9). An OGTT failed to suppress the levels of GH. An MRI showed a 28 × 25 × 12 mm pituitary tumour with invasion of the left cavernous sinus, suprasellar extension, and compression of the optic chiasma. She underwent an unsuccessful transsphenoidal surgery followed by a pterional craniotomy, with subtotal excision of the tumour. Histopathology showed a pituitary adenoma with immunoreactivity for GH and Ki-67 < 2%. Two months later, GH levels remained unsuppressed and IGF1 levels were 547 ng/mL (NR 117–329). Testing for other pituitary hormones revealed panhypopituitarism and hormone replacement therapy was initiated. An octreotide test trial resulted in a 63% decrease in GH levels. The patient began treatment with lanreotide 120 mg every 4 weeks and cabergoline 2 mg per week, which resulted in a partial response. Six months later, an MRI showed persistence of a large tumour residue. Eighteen months later, the patient underwent stereotactic radiosurgery and maintained treatment with lanreotide and cabergoline. Two years after radiosurgery, the patient showed clinical, biochemical and imaging improvement, and pharmacological treatment was gradually reduced to lanreotide 120 mg every 9 weeks and cabergoline 0.5 mg per week. At the last follow-up, 9 years after surgery, the patient stopped the treatment with lanreotide and maintained cabergoline 0.25 mg per week and remaining pituitary hormone replacement therapy. Subsequent testing showed a nadir postglucose GH level of 2.87 ng/mL, but with normal IGF1 levels. A head MRI showed residual tumour near the left cavernous sinus. There was no known family history of pituitary adenomas. Genetic analysis revealed an *AIP* heterozygous mutation (c.736G > T, p.Glu246*) in the patient and in her asymptomatic mother, who was referred for clinical, biochemical and MRI assessment.

## Discussion

The prevalence of pathogenic and likely pathogenic *AIP* variants in our cohort of patients with sporadic pituitary macroadenomas diagnosed under the age of 40 years was 1.8% (4/218). This represents a low prevalence when compared to that of other studies carried out in France (16/222, 7.2%) [11], Brazil (11/132, 8.3%) [18] and Australia (6/34, 17.6%) [19]. Other studies have used lower age cut-offs to analyse the prevalence of *AIP* mutations. A pan-European collaboration [12] analysed a cohort of 163 patients diagnosed with sporadic pituitary macroadenomas before the age of 30 years and reported an 11.7% (19/163) prevalence of *AIP* mutations. Likewise, other studies carried out in Turkey [20], Mexico [21], and Spain [22], in patients under 30 years, reported prevalences of *AIP* mutations of 9% (1/11), 7% (5/55), and 6% (9/148), respectively. In contrast, our study showed that only 3.4% (4/118) of Portuguese patients diagnosed under the age of 30 years had *AIP* mutations. Furthermore, only 5.0% (1/20) of our paediatric patients presented *AIP* mutations, which is also a prevalence lower than in other studies [11, 12, 18, 21, 22]. Interestingly, the reported prevalences in the UK (2/100, 2%, under 40 years) [23] and in Germany (2/82, 2.4%, under 30 years) [24] were closer to those observed in our study. More recently, a study in Poland comprising 131 patients with macroadenomas of all ages failed to identify any clearly pathogenic *AIP* mutations [25].

There may be several explanations for the observed differences across studied populations. First, there may be geographical and ethnic differences in the genetic makeup of the populations and founder effects that increase the frequency of certain mutations. Second, the overall clinical characteristics of the patients may differ between cohorts. For example, as *AIP* mutations occur more commonly in GH-secreting tumours [26], cohorts that are enriched for patients with acromegaly may present a higher prevalence of these mutations. Lastly, the criteria for classifying genetic variants as pathogenic often vary between studies. Several genetic variants reported as pathogenic or variants of uncertain significance (VUS) in earlier studies are now considered to be benign variants. For example, the benign missense (p.Arg16His and p.Ala299Val) and non-coding (c.*14C > A and c.*64G > A) variants, which we excluded from our study, were included in the prevalence data of other studies [11, 18]. The inclusion of VUS in prevalence studies is particularly problematic because their interpretation is often difficult and, over time, many are reclassified as benign variants [27]. Thus, it is possible that earlier studies have overestimated the prevalence of true pathogenic variants in young-onset pituitary macroadenomas.

We identified four *AIP* mutations, including two novel [c.158_165delGCCGGGCT (p.Ser53Thrfs*36) and c.736G > T (p.Glu246*)] and two that have been reported before in patients from other countries [c.241C > T (p.Arg81*) and c.343delC (p.Leu115Trpfs*41)] [7, 28]. All of these are frameshift or nonsense mutations that are expected to lead to a premature stop codon and consequently to the formation of a shorter protein or to nonsense-mediated decay [29]. Therefore, these are loss-of-function mutations that abolish protein domains that are important for the binding of AIP to its interaction partners [30].

The mutations identified in this study were all found in patients with GH-secreting adenomas. This is not unexpected, as *AIP* mutations have been found to be more prevalent in this tumour type [26]. Apart from the young age of the patients, the clinical course of the disease was not particularly unusual and the pituitary surgery was curative in all but one patient. Thus, in this limited group of patients, we were unable to confirm previous reports that suggested a more unfavourable outcome in patients with *AIP* mutations [1, 9].

None of the patients with *AIP* mutations reported a family history of pituitary adenomas. However, in two cases, the mutation was also identified in an asymptomatic parent who will now undergo clinical, biochemical and MRI assessment. It remains to be determined if these mutation carriers have clinically silent adenomas or incomplete penetrance of the *AIP* mutations. It has been estimated that *AIP* mutations are associated with a disease penetrance of only 20–25% [31] and this explains why family members who share the same mutation often do not express the disease. Nevertheless, our identification of patients with *AIP* mutations will allow for cascade genetic screening of family members, the identification of additional mutation carriers, the clinical screening of these mutation carriers, and an earlier diagnosis, treatment and, possibly, better long-term outcome of any existing pituitary tumours [10].

Our study has some limitations. First, we did not look for *AIP* gross deletions that have been reported before [24, 32, 33]. However, studies using Multiplex Ligation-dependent Probe Amplification (MLPA) in large cohorts of patients have not identified any gross gene deletions, indicating that these are unlikely to be frequent [11, 12]. Second, we did not look for mutations in other genes that may be associated with syndromic forms of pituitary adenomas, such as the multiple endocrine neoplasia type 1 (MEN1) gene [34]. Although we excluded patients with evidence of other coexisting endocrine tumours or other features suggestive of syndromic forms of pituitary adenomas, we cannot rule out the possibility of a pituitary adenoma being the first manifestation of an undiagnosed syndrome.

In conclusion, the prevalence of *AIP* mutations in our cohort is lower than that in previous reports, possibly because we used more stringent criteria to classify variants as pathogenic. No *AIP* mutations were found in patients diagnosed after the age of 30 years and this suggests that this age cutoff may be the most appropriate for *AIP* genetic testing in patients with apparently sporadic large pituitary adenomas. Finally, our identification of novel *AIP* mutations expands the known spectrum of *AIP* mutations and may contribute to the understanding of the pathogenesis of pituitary adenomas.

## Data Availability

The datasets generated during and/or analysed during the current study are available from the corresponding author on reasonable request.
